# Targeted Alpha-Particle Therapy: A Review of Current Trials

**DOI:** 10.3390/ijms241411626

**Published:** 2023-07-19

**Authors:** Albert Jang, Ayse T. Kendi, Geoffrey B. Johnson, Thorvardur R. Halfdanarson, Oliver Sartor

**Affiliations:** 1 Deming Department of Medicine, Tulane University School of Medicine, New Orleans, LA 70112, USA; 2 Department of Radiology, Mayo Clinic, Rochester, MN 55905, USA; 3 Department of Immunology, Mayo Clinic, Rochester, MN 55905, USA; 4 Department of Oncology, Mayo Clinic, Rochester, MN 55905, USA; 5 Department of Urology, Mayo Clinic, Rochester, MN 55905, USA

**Keywords:** targeted alpha therapy, radiopharmaceuticals, actinium-225, lead-212, astatine-211

## Abstract

Radiopharmaceuticals are rapidly developing as a field, with the successful use of targeted beta emitters in neuroendocrine tumors and prostate cancer serving as catalysts. Targeted alpha emitters are in current development for several potential oncologic indications. Herein, we review the three most prevalently studied conjugated/chelated alpha emitters (^225^actinium, ^212^lead, and ^211^astatine) and focus on contemporary clinical trials in an effort to more fully appreciate the breadth of the current evaluation. Phase I trials targeting multiple diseases are now underway, and at least one phase III trial (in selected neuroendocrine cancers) is currently in the initial stages of recruitment. Combination trials are now also emerging as alpha emitters are integrated with other therapies in an effort to create solutions for those with advanced cancers. Despite the promise of targeted alpha therapies, many challenges remain. These challenges include the development of reliable supply chains, the need for a better understanding of the relationships between administered dose and absorbed dose in both tissue and tumor and how that predicts outcomes, and the incomplete understanding of potential long-term deleterious effects of the alpha emitters. Progress on multiple fronts is necessary to bring the potential of targeted alpha therapies into the clinic.

## 1. Introduction

Radiopharmaceuticals now have a firmly established role in the treatment of multiple solid tumors, including thyroid cancer, and, more recently, selected neuroendocrine tumors and prostate cancer. Although beta-emitting isotopes have historically led the way, in 2013, the first alpha emitter was approved (^223^radium dichloride or ^223^Ra) after a phase III study (ALSYMPCA) indicated that overall survival could be prolonged with the use of this radionuclide [1]. While ^223^Ra was a conceptually strong step forward, radium targets only the bone stroma, and tumors outside osteoblastic bone metastases are not effectively treated by this agent. More recently, increased attention has been garnered by alpha emitters that can be targeted to receptors that are preferentially expressed on the surface of tumor cells. These radionuclides are typically attached to a targeting molecule, thus allowing tumors to be selectively targeted. In this review, we focus on the contemporary trials using targeted alpha therapy (TAT) while providing some overview of the isotopes themselves and some of the challenges that accompany this burgeoning but still immature and challenging field. Three isotopes will be focused on, based on their use in contemporary clinical trials. These isotopes include ^225^actinium (^225^Ac), ^212^lead (^212^Pb), and ^211^astatine (^211^At). One additional alpha-emitter isotope will be briefly noted, ^227^thorium (^227^Th); however, only one trial is currently using this isotope, and several trials using it have been discontinued. It is not apparent whether ^227^Th will be further developed in future human clinical trials. Thus, additional coverage herein is not warranted.

## 2. Notable Radioisotopes in Targeted Alpha Therapy

An increasing number of targeted alpha-emitting radioisotopes are being tested in current clinical trials. This section highlights select radioisotopes of interest (including information on their half-lives, selective information on decay chains, and potential applications). For additional details regarding the characteristics of these alpha emitters, including the full decay chains and production/purification methods, the authors recommend one of several well-written reviews [2,3,4].

^225^Ac has a half-life of 9.92 days as it degrades to ^221^Fr, emitting a 5.83 MeV alpha particle. ^221^Fr (half-life 4.9 min) subsequently emits another alpha particle and decays to ^217^At (half-life 32 ms), which emits an additional alpha particle to become ^213^Bi. ^213^Bi decays with a half-life of 46 min through alpha and beta emissions until the stable ^209^Bi is reached. The decay chain yields a total of four alpha particles (and two beta particles) and a cumulative energy of approximately 28 MeV, making ^225^Ac a particularly potent radionuclide [5]. Multiple chelation strategies for ^225^Ac are used, including DOTA and the more recently described macropa [6]. As with any decay chain with multiple alpha emissions, the recoil from the first alpha emission displaces the first daughter from the chelate, leaving subsequent daughters capable of diffusing from the targeting moiety [7]. Two isotopes in the decay chain, ^221^Fr and ^213^Bi, can potentially be imaged by single-photon emission tomography (SPECT) imaging. The decay of ^221^Fr emits a 218 keV gamma ray, and the ^213^Bi emits a 440 keV gamma ray. Such images are hampered by the fact that the typically administered dose of ^225^Ac (approximately 0.2 mCi) has low activity relative to imaging agents or targeted beta therapies, although imaging has now been reported in humans [8,9].

^211^At is a radiohalogen with metallic properties with a half-life of 7.2 h. It either undergoes alpha decay (42%) with a release of 5.9 MeV alpha leading to ^207^Bi (half-life 33 years), which undergoes an electron capture to result in stable ^207^Pb, or it first undergoes electron capture (58%), resulting in ^211^Po (half-life 0.53 ms), which in turn releases an alpha particle (7.5 MeV) to become ^207^Pb. Because there is only one alpha emission for ^211^At, that minimizes the concern of unpredictable dose localization and wandering radioactive daughters [10]. The decay branch to ^211^Po also produces X-rays (77 to 92 keV), which allows ^211^At to be imaged with SPECT [11]. Unlike the radiometals described elsewhere in this article, ^211^At can be covalently linked to a variety of molecules, including both small molecules and antibodies, potentially allowing a greater degree of flexibility for targeting compounds whose binding might be disrupted by chelate addition. That said, astatine chemistry is complex, and astatine-containing compounds may be prone to biologic instability. Astatine chemistry is understudied relative to others, in part, due to the lack of stable isotopes. The short half-life of ^211^At makes for challenging distribution models, particularly considering that the radionuclide must typically be linked to the targeting moiety and transported to the patient’s bedside prior to administration.

^212^Pb has a half-life of 10.6 h as it decays to ^212^Bi with the release of a beta particle. When bound to an appropriate chelate, it is not generally thought that this beta emission displaces the ^212^Bi from the chelate. ^212^Bi has a half-life of 61 min and ultimately degrades to a stable ^207^Pb after the release of both one alpha particle and one beta particle. This happens by one of two pathways: either ^212^Bi first releases an alpha particle of 6.1 MeV to become ^208^Tl and then a beta particle is released, or ^212^Bi first releases a beta particle to temporarily become ^211^Po before releasing an alpha particle of 8.8 MeV. The longer half-life of ^212^Pb compared with ^212^Bi allows time for the preparation of the radiopharmaceutical [12]. Common chelates used for ^212^Pb include DOTA and DOTAM (TCMC) [13]. ^212^Pb decays can potentially be imaged using a 239 keV gamma ray and X-rays from 75–91 keV; however, ^203^Pb (a “perfect pair” with ^212^Pb) is more readily imageable using a 279 keV gamma emission, given the higher doses of ^203^Pb that can be safely deployed. ^203^Pb has a half-life of 52 h, making its labeling, shipping, and study relatively manageable when focusing on SPECT imaging.

## 3. Contemporary TAT Clinical Trials

### 3.1. Trials Run by Commercial Pharmaceutical Companies

Many commercial pharmaceutical companies have begun TAT trials with cancer patients. This section provides an overview of the most recent recruiting/active and planned clinical trials. Some trials have reported promising preliminary data, likely leading to upcoming phase III trials. Several targets are of clear interest, such as prostate-specific membrane antigen (PSMA) in prostate cancer and somatostatin receptor 2 (SSTR2) in neuroendocrine tumors. Other target proteins are being explored in various solid and hematologic malignancies. These commercial trials are summarized in Table 1.

Weill Cornell Medicine has studied ^225^Ac-J591 in different combinations and in different stages of prostate cancer. The J591 monoclonal antibody binds to the extracellular domain of PSMA at a site distinct from smaller agents such as the well-studied PSMA-617 [14]. The first phase I study (NCT03276572) involved 32 men with heavily pre-treated mCRPC, including patients with prior ^223^Ra and ^177^Lu-PSMA treatment, who received a single dose of ^225^Ac-J591. A dose of up to 93.3 kBq/kg (2.52 µCi/kg) was tolerated, and 12 out of 32 patients had a PSA decline >50%. Median progression-free survival (PFS) was 7.2 months (95% CI, 4.6-NR), and median overall survival was 10.9 months (95% CI, 7.6–21.1) after a single dose [15]. Based on this successful study, a phase I/II study (NCT04506567) aims to determine the optimal dose of ^225^Ac-J591 for 105 patients with mCRPC. A dose-fractionated cohort includes patients who will receive a dose of ^225^Ac-J591 on days 1 and 15, and a multiple-dose cohort includes patients who will receive ^225^Ac-J591 every six weeks for up to four cycles [16]. Primary endpoints include the recommended phase 2 dose (RP2D), cumulative maximum tolerated dose (MTD), and dose-limiting toxicities (DLT). The estimated primary completion date is June 2025. Another phase I study (NCT04576871) is enrolling up to 18 participants with progressive mCRPC to receive a first dose of ^225^Ac-J591 at 90 kBq/kg (2.4 µCi/kg); if the dose is well tolerated, subjects will receive a second dose at least three months after the initial dose. This study’s estimated primary completion date is December 2023.

Combinations of ^225^Ac-J591 with other agents in the mCRPC setting include ^177^Lu-PSMA-I&T (NCT04886986) and pembrolizumab with ARPI (NCT04946370). In the phase I/II study of ^225^Ac-J591 with ^177^Lu-PSMA-I&T, up to 18 patients are to be enrolled in phase I to receive either 30, 35, or 40 kBq/kg (0.81, 0.95, or 1.1 µCi/kg) of ^225^Ac-J591 in a dose-escalation setting every eight weeks for up to two cycles along with the co-administration of 6.8 GBq (184 mCi) of ^177^Lu-PSMA-I&T every eight weeks for up to two cycles to determine the optimal phase II dose, and then up to 24 patients will receive this dose with 6.8 GBq (184 mCi) of ^177^Lu-PSMA-I&T every eight weeks for up to two cycles in the phase II setting. Primary endpoints include RP2D, cumulative MTD, DLT, and the proportion of patients with PSA decline. The early results from this ongoing study were released at the 2023 ASCO Annual Meeting [17]. Seventeen out of 18 patients (94%) showed PSA decline, and 9/18 (50%) had PSA50 (PSA decline more than 50%). DLT were observed in 2/6 patients treated with 40 kBq/kg (1.1 µCi/kg) ^225^Ac-J591, whereas DLT were not observed in the other two cohorts treated with 30 and 35 kBq/kg (0.81 and 0.95 µCi/kg) ^225^Ac-J591. The estimated primary completion is December 2024. In the phase I/II study of ^225^Ac-J591 with pembrolizumab and ARPI, there are 76 patients enrolled. In the phase I setting, patients will receive ^225^Ac-J591 at either 65 or 90 kBq/kg (1.8 or 2.4 µCi/kg) in addition to pembrolizumab and ARPI, and the optimal dose for phase II will be determined. In the phase II setting, patients will be randomized to receive either one dose of ^225^Ac-J591 with ARPI and pembrolizumab or ARPI and pembrolizumab alone in a 1:1 ratio. Early results from the phase I portion of the study were presented at the 2023 ASCO GU Meeting [18]. All 12 patients showed PSA decline, with 6/12 (50%) showing more than a 50% decline. With more than 6 months follow-up, 4/12 (33%) remained progression-free. Seven (58%) showed an unexpected cytokine release syndrome (CRS) 1–2 weeks following treatment that was characterized by morbilliform rash, fever, and low blood counts. The estimated primary completion date is June 2025.

In the hormone-sensitive setting, ACTION (NCT05567770) is a phase I study enrolling 24 patients with recurrent PSMA-positive hormone-sensitive prostate cancer. It is composed of two cohorts: cohort 1 has oligometastatic disease, and cohort 2 has high volume (≥5 metastases). The patients in cohort 1 will receive ^225^Ac-J591 with stereotactic body radiation therapy (SBRT), and the patients in cohort 2 will receive ^225^Ac-J591 with androgen-deprivation therapy (ADT). The primary endpoints are DLT and MTD. The anticipated study start date is June 2023, and the estimated primary completion date is January 2025.

Fusion Pharmaceuticals Inc. has been studying alpha emitters against several targets, including insulin-like growth factor 1 receptor (IGF-1R), neurotensin receptor 1 (NTSR1), and the recently acquired well-known small molecule PSMA I&T. FPI-1434 is a humanized monoclonal antibody that binds to the external domain of IGF-1R. A phase I/II trial (NCT03746431) is evaluating ^225^Ac-FPI-1434 in locally advanced and metastatic solid tumors that are positive for IGF-1R (determined by demonstrating the uptake of ^111^In-FPI-1547, a radioimmuno-imaging agent, and the anti-IGF-1R antibody FPI-1175) [19]. Phase I is a dose-escalation study beginning with 10 kBq/kg (0.3 µCi/kg) and increasing up to 120 kBq/kg (3.2 µCi/kg) in a single injection or multi-dose injection every 42 days, with primary endpoints including DLT and adverse events. Phase II will evaluate anti-tumor activity and the objective response rate per RECIST 1.1 criteria. A total of 253 participants are enrolled, with estimated primary completion in June 2023.

Another phase I study (NCT05605522) enrolling 42 patients is evaluating ^225^Ac-FPI-2059 in metastatic solid tumors that express NTSR1. Six solid tumors are prioritized: head and neck squamous cell carcinoma, pancreatic ductal adenocarcinoma, colorectal carcinoma, gastric carcinoma, Ewing sarcoma, and neuroendocrine prostate cancer. The radioimmuno-imaging agent is ^111^In-FPI-2058, and ^225^Ac-FPI-2059 will be infused every 56 days for up to four cycles. The primary outcome measures include adverse events, MTD, and radiation dose. The estimated primary completion date is June 2025.

Fusion Pharmaceuticals has also acquired the phase II program TATCIST (NCT05219500) of ^225^Ac-PSMA-I&T (FPI-2265) from RadioMedix, Inc. TATCIST is enrolling 100 patients with mCRPC, both naïve and pre-treated with ^177^Lu-based PSMA pharmaceuticals, to receive ^225^Ac-PSMA-I&T at 8-week intervals for four cycles, starting at 100 kBq/kg (2.7 µCi/kg) with dose de-escalation down to 50 kBq/kg (1.4 µCi/kg) in those with a good PSA response. The primary endpoint of the study is the treatment efficacy, defined as PSA50, with ^225^Ac-PSMA-I&T. The estimated primary completion date is December 2023.

Telix Pharmaceuticals is preparing patients for TAT, with clinical trials currently in the imaging phase. If these antibodies that are conjugated to an imaging isotope are successful, the antibodies will be conjugated to ^225^Ac and tested in patients in future clinical trials. CUPID (NCT04726033) is a phase I trial enrolling 15 patients with prostate cancer that is evaluating the adverse events, pharmacokinetics, biodistribution, and radiation dosimetry of TLX592 (targeting PSMA) using ^64^Cu as the imaging agent. Pending the success of this trial, TAT using ^225^Ac conjugated to TLX592 will be tested in a future trial in prostate cancer patients. TLX592 is a derivative of J591 designed to have faster blood clearance. In an analogous situation, PERTINENCE (NCT04897763) is a phase I trial enrolling six participants with non-muscle invasive bladder cancer to study the effects of the PET imaging agent ^89^Zr-DFO-girentuximab (TLX250-CDx), which will be administered via intravesical infusion. Girentuximab targets carbonic anhydrase IX, which is a cell-surface protein highly expressed on many cancer cells, including in the kidney and bladder. If successful, a TAT clinical trial with ^211^At conjugated to girentuximab will be implemented.

Bayer has one active phase I clinical trial involving ^227^Th-labeled radioimmunoconjugates; however, ^227^Th prostate trials are noted as not being actively recruiting at this time. NCT04147819 is enrolling 14 patients with HER2-expressing tumors (not limited to breast, gastric, and gastroesophageal cancers) who have failed standard therapies to receive BAY2701439 (^227^Th attached to the antibody portion of trastuzumab) in a dose-escalating fashion. Both trials will evaluate adverse events and MTD.

Bayer acquired Noria and PSMA Therapeutics Inc. in June 2021, which allows for significant expansion of its TAT program by adding ^225^Ac small molecules targeting PSMA to its options. Noria studied different small molecules that could target PSMA in LNCaP xenograft models. These molecules bind to albumin, which prolongs their half-life relative to non-albumin-binding small molecules, and in animal models, such an approach can increase the dose delivered to the tumor relative to the kidney [20]. RPS-072 was one molecule that was found to provide increased tumor activity with decreased kidney activity compared with other small molecules, suggesting that RPS-072 could provide increased delivery of a radioisotope to a tumor and making it a promising candidate for future clinical trials [21].

Similarly, Isotope Technologies Munich SE has developed a new class of PSMA radioligands using ibuprofen-like moieties as the albumin binder to improve pharmacokinetic properties, better tumor uptake, and thus better tumor-killing efficiency, while allowing for efficient clearance from the bloodstream to protect healthy tissues. Deberle and colleagues [22] evaluated ^177^Lu-Ibu-diaminobutyric acid (DAB)-PSMA in the preclinical setting and found that it was able to accumulate 40% higher in the tumor compared with ^177^Lu-PSMA-617 while being quickly cleared in the blood and kidneys in murine models. Another study found the S-isomer of ibuprofen (Sibu) to bind better to plasma proteins than the R-isomer of ibuprofen [23]. Busslinger and colleagues [24] compared ^225^Ac-SibuDAB to ^225^Ac-PSMA-617 in murine models and found ^225^Ac-SibuDAB to be more effective at destroying PSMA-positive tumor xenografts, although ^225^Ac-SibuDAB also had higher retention in normal tissues. The study suggests that ^225^Ac-SibuDAB may enter early-phase clinical trials soon.

AdvanCell Isotopes Pty Limited is evaluating ^212^Pb-ADVC001 (targeting PSMA) in the phase 1/2 trial TheraPb (NCT05720130) in 18 patients with PSMA-positive mCRPC with no prior exposure to ^177^Lu-PSMA-based therapies. Patients will be divided into four cohorts receiving doses of 60, 90, 120, and 150 MBq (1.6, 2.4, 3.2, and 4.1 mCi) for a maximum of four cycles every six weeks. The primary outcome is the RP2D, and the estimated primary completion date is December 2023.

Novartis Pharmaceuticals is evaluating ^225^Ac-PSMA-617 in the phase I trial AcTION (NCT04597411) (not to be confused with ACTION, NCT05567770), which is enrolling 60 patients with PSMA-positive prostate cancer. The patients will be enrolled into three different cohorts: without prior exposure to ^177^Lu-PSMA-617 or ^177^Lu-PSMA-I&T and without prior chemotherapy or ARPI, with prior chemotherapy and an ARPI but no prior exposure to ^177^Lu, and with prior exposure therapy to ^177^Lu. The primary outcome is the RP2D, and the estimated primary completion date is October 2023. Phase III plans are under consideration.

Janssen Pharmaceuticals has developed ^225^Ac-DOTA-h11B6 (JNJ-69086420) for a phase I trial (NCT04644770) in 100 patients with mCRPC with prior ARPI exposure [25]. h11B6 is a monoclonal antibody that binds to human kallikrein-2 (hK2), a prostate cancer tumor marker [26]. The subjects received one or more escalating doses of ^225^Ac-DOTA-h11B6. Primary outcome measures include adverse events and DLT in order to determine an optimal phase II dose. This trial is currently ongoing.

Radiomedix, Inc. and Orano Med LLC are collaborating to evaluate ^212^Pb-DOTAMTATE (AlphaMedix^TM^) in patients with unresectable, metastatic neuroendocrine tumors (NETs) that express somatostatin receptor type 2 (SSTR2). DOTAM (similar in structure to DOTA) is the bifunctional metal chelator, and TATE is the SSTR-targeting peptide [27]. The phase I trial (NCT03466216) enrolled 33 patients with SSTR2-positive NETs with no prior peptide–receptor radionuclide therapy (PRRT). Preliminary results of this trial were reported by Delpassand and colleagues [27] in 2022 with data from 20 subjects. Patients received increasing doses, and the RP2D regimen was determined to be four cycles of 2.50 MBq/kg/cycle (67.6 µCi/kg/cycle) of ^212^Pb-DOTAMTATE, which 10 patients tolerated well. Eight of these 10 patients had an objective radiographic response, including one patient with a complete response. There were no serious treatment-related adverse events that were related to the study drug. The phase II trial (NCT05153772) is evaluating ^212^Pb-DOTAMTATE in 34 patients using this 67.6 µCi/kg dose per cycle. Primary outcome measures will be the objective response rate measured by RECIST 1.1 and adverse events. The estimated study completion date is December 2023.

Similarly, RayzeBio, Inc. is investigating RYZ101, which delivers ^225^Ac to NETs expressing SSTR2. The phase Ib/III clinical trial ACTION-1 (NCT05477576) is enrolling 218 patients with SSTR-positive gastroenteropancreatic (GEP) NETs that have progressed with prior ^177^Lu-DOTATATE or ^177^Lu-DOTATOC therapy. In the phase Ib part, a recommended phase III dose will be determined as patients receive up to four infusions of RYZ101 every eight weeks. In the phase III part, RYZ101 will be compared with the standard of care (either everolimus, sunitinib, or a somatostatin analog of either octreotide 60 mg monthly or lanreotide 120 mg every other week), with the primary endpoint of PFS based on RECIST 1.1. The part 1 (phase Ib) results were presented at the 2023 ASCO Annual Meeting [28]. RYZ101 was well tolerated at 120 kBq/kg (3.2 µCi/kg), which was declared the recommended phase 3 dose. Part 2 (Phase III) commenced in May 2023. RYZ101 is also being evaluated in combination with the standard-of-care regimen of carboplatin, etoposide, and atezolizumab for 31 patients with extensive-stage small-cell lung cancer expressing SSTR in a phase Ib trial (NCT05595460), with primary outcome measures of safety, tolerability, and RP2D. The estimated primary completion date is April 2024.

Orano Med LLC, in addition to collaborating with RadioMedix to treat NETs, is evaluating TAT in advanced solid tumors expressing gastrin-releasing peptide receptor (GRPR). A phase I trial (NCT05283330) is evaluating ^212^Pb-DOTAM-GRPR1 in 50 patients with recurrent or metastatic GRPR-expressing tumors. Patients are grouped in up to four cohorts, and dose expansion will be evaluated, with a maximum total dose of 24 mCi over four cycles. The primary outcome measure will be to determine an RP2D. The estimated primary completion date is August 2024.

Actinium Pharmaceuticals has been evaluating ^225^Ac-lintuzumab (^225^Ac-HuM195) in hematologic malignancies. Lintuzumab is a humanized monoclonal antibody that targets CD33, which is expressed in a subset of lymphoid neoplasms such as acute myeloid leukemia (AML) as well as multiple myeloma. In a phase I/II trial (NCT02575963), 40 elderly patients (median age 75 years) with untreated AML received ^225^Ac-linzutumab of either 2.0 μCi/kg or 1.5 μCi/kg (dose reduced from 2.0 to 1.5 μCi/kg due to the incidence of grade 4 thrombocytopenia seen in phase I), and 69% of patients receiving 2.0 μCi/kg had an objective response while 22% receiving 1.5 μCi/kg had an objective response [29]. The study was closed to further accrual due to the adverse event profile, but it showed that ^225^Ac-lintuzumab warranted more evaluation. Other trials with ^225^Ac-linzutumab combined with other agents are underway in the refractory/relapsed AML setting. In a phase I trial (NCT03441048) for patients with refractory/relapsed AML, Actinium Pharmaceuticals is collaborating with the Medical College of Wisconsin to assess the combination of ^225^Ac-linzutumab with cladribine, cytarabine, filgrastim, and mitoxantrone (CLAG-M). Preliminary results reported at the 2022 American Society of Hematology Annual Meeting showed that ^225^Ac-linzutumab at 0.75 μCi/kg combined with CLAG-M was feasible, safe, and had a high response rate for treated patients [30]. A phase I/II trial (NCT03867682) is evaluating the combination of ^225^Ac-linzutumab and venetoclax, with primary outcome measures of MTD (phase I) and overall response (phase II). Another phase I/II trial (NCT03932318) to evaluate the triplet regimen of ^225^Ac-linzutumab, venetoclax, and azacitidine, with primary outcome measures of MTD (phase I) and overall response (phase II), has not started recruiting patients yet.

Perspective Therapeutics, Inc. (previously known as Viewpoint Molecular Targeting, Inc.) plans to evaluate ^212^Pb-VMT01. VMT01 targets the melanocortin subtype-1 receptor (MC1R) overexpressed on melanoma cells [31]. In an upcoming phase I/IIa trial (NCT05655312), 52 patients with previously treated unresectable and metastatic melanoma and a positive MC1R imaging scan with either ^203^Pb-VMT01 or ^68^Ga-VMT02 will receive ^212^Pb-VMT01. The first phase includes a dose escalation in 32 patients receiving up to three infusions eight weeks apart to determine MTD. A second phase will include 20 patients receiving further dose expansion. Primary outcome measures include rates of adverse events and lab abnormalities, DLT, and ORR per RECIST 1.1.

Another agent under clinical investigation by Perspective Therapeutics is VMT-α-NET, which targets SSTR2. Currently, a phase I study (NCT05111509) is using the imaging agent ^203^Pb-VMT-α-NET for somatostatin receptor imaging of neuroendocrine tumors in 20 patients. The estimated primary completion date is June 2023. A forthcoming phase I/IIa TAT trial (NCT05636618) with ^212^Pb-VMT-α-NET in patients with SSTR2-positive tumors is expected to activate soon. In this trial, a dose-escalation phase I part will establish an RP2D followed by a phase IIa dose-expansion cohort evaluating efficacy using the RP2D.

POINT Biopharma Global Inc. has released preclinical data on ^225^Ac-PNT2001, which targets PSMA. Data presented at the 35th Annual Congress of the European Association of Nuclear Medicine showed increased internalization of ^225^Ac-PNT2001 by tumor cells in vitro, more precise tumor targeting with decreased kidney uptake and high tumor retention in murine models, and good therapeutic activity after one dose in murine models, suggesting this ^225^Ac-PNT2001 will advance to clinical evaluation [32].

POINT Biopharma is also studying PNT6555, which targets fibroblast activation protein-α (FAP-α). FAP-α which is highly expressed on a wide range of solid tumors including breast, ovarian, colorectal, pancreatic, and lung [33]. Currently, ^177^Lu-PNT6555 is under dose-escalation investigation in a phase I trial (NCT05432193) for patients with FAP-avid solid tumors, with estimated primary completion in December 2023. There are upcoming plans for evaluating ^225^Ac-PNT6555 against FAP-positive tumors as well.

### 3.2. Investigator-Initiated Trials

In addition to commercially led TAT trials, investigator-initiated trials are also ongoing, which are summarized in Table 2. Osaka University in Japan is studying TAH-1005 (^211^At) in a dose-escalation phase I study (NCT05275946) involving 11 patients with differentiated thyroid cancer refractory to standard treatment. ^211^At is taken up by thyroid cells via the sodium iodide symporter because of its chemical similarities to iodine. Patients will receive one infusion of TAH-1005. Primary endpoints include adverse events and DLT.

Fukushima Medical University in Japan is studying ^211^At-meta-astatobenzylguanidine (MABG) in patients with malignant pheochromocytoma or paraganglioma. ^211^At-MIBG was demonstrated in preclinical studies to have anti-tumor effects in a pheochromocytoma mouse model [34]. MABG targets adrenergic tissue through the norepinephrine transporter. The dose escalation trial will determine safety, MTD, and an RP2D [10].

Fred Hutchinson Cancer Center in Seattle, Washington is studying ^211^At in hematopoietic stem cell transplantation using the antibodies BC8-B10 (targeting CD45, which is expressed on almost all hematopoietic cells except for mature erythrocytes [35]) and OKT10-B10 (targeting CD38, which is highly expressed in plasma cells in multiple myeloma [36]), with the goal of decreasing potential toxicity by decreasing the intensity of myeloablative regimens, which older patients and those with multiple comorbidities are unable to tolerate. A phase I/II trial (NCT04083183) is evaluating ^211^At-BC8-B10 along with other traditional bone marrow-depleting agents and total body irradiation for 40 patients receiving hematopoietic stem cell transplants, and the patients will be followed up for up to five years. Similarly, another phase I/II trial (NCT03670966) is using ^211^At-BC8-B10 along with other traditional agents for 30 patients with relapsed/refractory acute leukemia or myelodysplastic syndrome before stem cell transplantation. Two phase I trials, NCT04579523 and NCT04466475, are each enrolling 30 patients with multiple myeloma to receive ^211^At-OKT10-B10 in combination with other traditional agents before stem cell transplantation, with MTD as the primary outcome.

City of Hope in Duarte, California is currently recruiting patients for two phase I TAT trials. NCT05204147 is enrolling 20 patients with malignancies that express carcinoembryonic antigen (CEA) to receive one infusion of ^225^Ac-DOTA-M5A. M5A is a humanized monoclonal antibody that binds to CEA [37]. Patients will be followed for up to six months. Primary outcome measures are adverse events and MTD. NCT05363111 is enrolling 15 patients with relapsed/refractory multiple myeloma to receive infusions of daratumumab and ^225^Ac-DOTA-daratumuab along with ^111^In-DOTA-daratumumab as the imaging agent over a four-hour period, with follow-up over the next twelve months. Primary outcome measures are DLT and MTD.

## 4. Challenges and Opportunities

It is well known that supply chains with radiopharmaceuticals can be problematic, and these supply chains are beyond the scope of this review. Isotopes used for cancer therapy decay relatively rapidly, are relatively expensive to make, and are heavily regulated. Their production, purification, chelation, shipping, and administration must be highly choreographed to be successful. Any disruption in the supply chain can leave a patient suffering without a dose. Alpha emitters are relatively new; thus, the technologies related to their production and the needed infrastructure are still emerging.

The emission of an alpha particle from a radionuclide in a radiopharmaceutical causes the release of the daughter nuclide from the chelate or conjugate bond because the recoil energy from the alpha emission exceeds the energy of the chelate/parent radionuclide bond. Isotopes such as ^225^Ac function as an “in vivo alpha-generator” and release multiple additional alpha particles that might or might not be confined to the tumor. Some studies have implicated ^213^Bi (half-life 46 min) as being a particularly problematic issue, as this isotope may be cleared by the kidneys. Different solutions have been proposed to combat this issue, such as using nanoparticles for encapsulating the radioisotope, using radioisotopes that either give off just one alpha particle before stabilizing (^212^Pb or ^211^At), and/or injecting radionuclides directly into the tumor tissue [38].

Since there is a known 5-year risk of renal failure in 5% of patients subjected to bilateral renal external beam radiation doses of 23 Gy, some have considered the kidneys to be the target organ (the organ which will likely show toxicity first), with a “tolerance dose” limit of 23 Gy [39]. This is despite the growing evidence that radiation in the form of a radiopharmaceutical spreads out over long periods of time and has far less renal toxicity than external beam radiation. Should a 23 Gy limit continue to be imposed, this could be a major barrier to the needed development of TAT in selected settings. For example, prostate cancer patients treated with six cycles of 7.4 GBq (200 mCi) ^177^Lu-PSMA-617 will have received an estimated dose just shy of 23 Gy, leaving no room for other radiation exposure. The absorbed dose estimate for alpha particles is extremely difficult to assess (even with accurate imaging), and radiobiologic equivalents of alpha particles, compared with other forms of radiation, remain somewhat conjectural. Furthermore, since alpha particles travel very small distances, their effects will be different than other forms of radiation, and the microscopic tissue distribution of these new TATs are currently difficult to assess. Microdosimetric calculations have been performed by some investigators, and these studies have concluded that capillary lumen containment versus the capillary traversal probability can significantly alter tumor-absorbed dose calculations [40]. Of note, anti-vascular targeted alpha therapy is potentially of interest. Taken together, external beam photons and intravenously administered targeted alpha particles are not readily comparable and attempts to extrapolate one to another are fraught with uncertainty.

Antibodies or albumin-binding small molecules may mitigate renal clearance, thereby diminishing renal isotopic exposure. Certain prodrugs have been demonstrated in model systems to have diminished uptake of PSMA-binding in the kidneys [41,42], thus providing a potential path forward in terms of mitigating renal exposure to radiation.

Damage to vital organs and tissues, including the bone marrow, cannot be ignored. From the marrow perspective, both acute and chronic toxicities may be of concern. Acute alterations are easily verifiable and assessed, but chronic effects require long-term follow-up as marrow dysplasia, leukemias, aplasia, and disruption of hematopoietic precursors may not manifest until years after radiation exposure [43,44].

Less discussed but apparent, alpha particles can damage extranuclear parts of cells, including cell membranes [45]. Bystander effects of alpha therapy can increase non-targeted tumor-cell killing, which has been previously shown with ^223^Ra in murine models [46], and this bystander effects can trigger immune responses that may target distant tumors as well [47]. The bystander effect is promising from the anti-tumor perspective but may be problematic from a toxicity perspective.

Interactions between alpha particles and the immune system are complex. Some data clearly indicate that radiation may damage the immune system, whereas other data suggest synergistic interactions that could be exploited for therapeutic gain [48]. Clearly, there are both challenges and opportunities in the alpha particle/immune interaction arena, and only carefully controlled human studies will determine the proper path forward (which may vary from tumor to tumor).

Though everybody advocates a cautious approach to drug development, clinical data obtained under careful dose escalations to assess MTD in a classical manner are likely the only way to properly understand the relationships between dose and toxicity. Once a toxicity is identified for a given TAT, then imaging-based dosimetry can be used to help predict the toxicity for that specific TAT, perhaps even in a personalized patient-specific manner that considers the distribution and volume of metastases. Phase I trials are typically conducted in heavily pre-treated patients with high burden of disease, and long-term follow-up will likely be limited in the initial exploratory prospective studies of these agents.

Dosimetry (calculating the dose of radiation delivered to organs and tumors) based on imaging with beta emitters is relatively straightforward. However, dosimetry is difficult with alpha emitters given that most of the radionuclides that emit alpha particles emit no or very few useful gamma rays or positrons for imaging when given at safe doses; thus, they are not well suited for imaging with SPECT or PET. Several innovative approaches may warrant further investigation. Results of a recently published article by Bobba and colleagues [49] showed potential for the ^134^Ce/^134^La pair as a PET imaging surrogate for the distribution of ^225^Ac-based radioligand therapies. Another possible solution is the use of ^149^terbium. This isotope emits both an alpha particle and a positron; however, its half-life is 4.1 h, hampering issues related to its production/administration, and the number of positrons emitted may only allow for the imaging of larger structures [50]. Often, the best option is to use an imaging radiopharmaceutical that is structurally similar, but not identical, to the TAT molecule to make a best guess at the biodistribution of the therapy. This is fraught with uncertainties but may prove better than not using imaging at all. One can at least argue that imaging in such a way allows for the selection of patients with cancer cells that express the TAT target. An additional solution lies in a binding moiety to which more than one chelator is attached: one capable of containing a positron emitter and the other, an alpha emitter. The first such successful molecules have been described in preclinical studies chelating ^212^Bi, an alpha emitter, and ^68^Ga, a positron emitter [51]. This approach did not move forward to clinical trials. Next-generation versions (so called “Alpha-PET”) using double chelators for ^212^Pb and ^64^Cu, ^225^Ac and ^89^Zr, or ^225^Ra and ^89^Zr have been developed and are completing preclinical testing [52]. When using such an approach with a longer-lived positron emitter such as ^64^Cu or ^89^Zr, the destination of the alpha emitter (assuming it remains in the chelate) can be quantitatively tracked. Similar approaches could be used for SPECT imaging with agents such as ^203^Pb, ^123^I, ^99m^technectium or ^111^In. “Perfect pairs” such as ^203^Pb and ^212^Pb can also clarify the isotopic distribution as they can utilize identical chelators and binders.

Another issue recently shown to be feasible in animal models is the ability to image DNA damage in vivo using positron-emitting poly(adenosine diphosphate-ribose) polymerase (PARP) inhibitors. Rucaparib [53], olaparib [54], and PARPZ [55] have been labeled with positron emitters such as ^18^F, and DNA damage can be monitored in vivo. The most direct application uses ^225^Ac-PSMA-617 to treat xenografts in tumor-bearing mice and imaging with ^18^F-PARPZ to monitor the DNA damage [55]. Such technologies may hypothetically be used to assess alpha-induced DNA effects in humans and thus may be useful in the early determination of the response and resistance to a variety of DNA-damaging agents. Applications of this technology in humans receiving TAT have not been reported.

Alpha-emitting therapies are potentially capable of offering important new therapeutic advances and of causing long-term deleterious side effects. Clinical trials will determine their clinical utility and how to optimally use these important new therapies. Dosimetry optimization, combination therapies, and patient/tumor selection will likely play pivotal roles going forward. The acute effects of radiation are relatively easy to measure, but chronic effects are somewhat unpredictable and may not appear for years or may never be apparent in advanced cancer patients because their longevity is limited. Long-term follow-up after human ^223^Ra therapies has been mandated by the FDA, and it is likely that long-term follow-up will be mandated after all TAT therapies as well. Interestingly, the data to date indicates little long-term sequelae post-^223^Ra [56], but given the limited life span of these patients, conclusions are necessarily incomplete. Some believe that secondary cancers, perhaps the worst long-term side effect of radiation exposure, may prove to be less common with alpha emitters than with beta emitters because of how they damage cells. Beta emitters predominantly cause simple lesions such as base damage and single-strand DNA breaks, which give the cells a chance to repair their DNA and survive. When the repair is inaccurate, it can lead to mutations, which can lead to secondary cancers. Alpha emitters cause both simple and complex lesions, including double-strand DNA breaks, from which the cells often do not survive. Time will tell if targeted alpha emitters are associated (or not) with secondary cancers. More long-term follow-up data are needed for all the alpha emitters as they become more prevalently used in human studies.

## Figures and Tables

**Table 1:** Current active and recruiting clinical trials with commercial sponsors using targeted alpha therapy.

Trial Number	Alpha Particle	Target	Agent(s)	Setting	Primary Outcome Measures
Cornell					
NCT03276572	^225^Ac	PSMA	^225^Ac-J591	mCRPC treated with prior ARPI	DLT, MTD
NCT04506567	^225^Ac	PSMA	^225^Ac-J591	mCRPC treated with prior ARPI	DLT, MTD, RP2D
NCT04576871	^225^Ac	PSMA	^225^Ac-J591	mCRPC treated with prior ARPI	DLT
NCT04886986	^225^Ac	PSMA	^225^Ac-J591 with ^177^Lu-PSMA-I&T	mCRPC treated with prior ARPI	DLT, MTD, RP2D, PSA decline
NCT04946370	^225^Ac	PSMA	^225^Ac-J591 with pembrolizumab and ARPI	mCRPC treated with prior ARPI	DLT, RP2D, response rate
NCT05567770	^225^Ac	PSMA	^225^Ac-J591	mHSPC	DLT, MTD
Fusion Pharmaceuticals					
NCT03746431	^225^Ac	IGF-1R	^225^Ac-FPI-1434	IGF-1R-positive solid tumors refractory to standard therapies	AE, DLT, ORR
NCT05605522	^225^Ac	NTSR1	^225^Ac-FPI-2059	NTSR1-positive solid tumors refractory to standard therapies	AE, MTD
NCT05219500	^225^Ac	PSMA	^225^Ac-FPI-2265 (PSMA-I&T)	mCRPC with prior ARPI	PSA50, safety
Bayer					
NCT04147819	^227^Th	HER2	BAY2701439	HER2-positive solid tumors refractory to standard therapies	AE, ORR
AdvanCell					
NCT05720130	^212^Pb	PSMA	^212^Pb-ADVC001	mCRPC with prior ARPI and no prior exposure to ^177^Lu	RP2D
Novartis					
NCT04597411	^225^Ac	PSMA	^225^Ac-PSMA-617	mCRPC	RP2D
Janssen					
NCT04644770	^225^Ac	hK2	^225^Ac-DOTA-h11B6 (JNJ-69086420)	mCRPC with prior ARPI	AE, DLT
Radiomedix and Orano Med					
NCT03466216	^212^Pb	SSTR2	^212^Pb-DOTAMTATE	SSTR2-positive neuroendocrine tumors refractory to standard therapies	DLT, MTD
NCT05153772	^212^Pb	SSTR2	^212^Pb-DOTAMTATE	SSTR2-positive neuroendocrine tumors refractory to standard therapies	ORR, AE
RayzeBio					
NCT05477576	^225^Ac	SSTR2	RYZ101	SSTR2-positive gastroenteropancreatic neuroendocrine tumors with prior ^177^Lu therapy	RP3D, PFS
NCT05595460	^225^Ac	SSTR2	RYZ101 with carboplatin, etoposide, and atezolizumab	SSTR2-positive extensive-stage small-cell lung cancer	RP2D, safety, tolerability
Orano Med					
NCT05283330	^212^Pb	GRPR1	^212^Pb-DOTAM-GRPR1	GRPR1-positive solid tumors refractory to standard therapies	RP2D
Actinium Pharmaceuticals					
NCT03441048	^225^Ac	CD33	^225^Ac-lintuzumab with cladribine, cytarabine, filgrastim, and mitoxantrone	Relapsed/refractory AML	DLT, MTD, AE, OS
NCT03867682	^225^Ac	CD33	^225^Ac-lintuzumab with venetoclax	Relapsed/refractory AML	MTD, overall response

Abbreviations: PSMA, prostate-specific membrane antigen; mCRPC, metastatic castration-resistant prostate cancer; ARPI, androgen-receptor pathway inhibitor; DLT, dose-limiting toxicities; MTD, maximum tolerated dose; RP2D, recommended phase 2 dose; mHSPC, metastatic hormone-sensitive prostate cancer; IGF-1R, insulin-like growth factor 1 receptor; AE, adverse events; ORR, objective response rate; NTSR1, neurotensin receptor 1; FGFR3, fibroblast growth factor receptor 3; PSA50, PSA decline by at least 50%; hK2, human kallikrein 2; SSTR2, somatostatin receptor type 2; PFS, progression-free survival; RP3D, recommended phase 3 dose; GRPR1, gastrin-releasing peptide receptor; AML, acute myeloid leukemia; OS, overall survival.

**Table 2:** Current active and recruiting investigator-initiated clinical trials using targeted alpha therapy.

Trial Number	Alpha Particle	Target	Agent(s)	Setting	Primary Outcome Measures
NCT05275946	^211^At	Thyroid tissue	TAH-1005	Differentiated thyroid cancer refractory to standard therapies	AE, DLT
N/A	^211^At	Norepinephrine transporter	^211^At-meta-astatobenzylguanidine	Pheochromocytoma and paraganglioma	Safety, MTD, phase 2 dose
NCT04083183	^211^At	CD45	^211^At-BC8-B10	Hematopoietic stem cell transplant regimen for non-malignant hematologic diseases	Graft rejection
NCT03670966	^211^At	CD45	^211^At-BC8-B10	Hematopoietic stem cell transplant regimen for malignant hematologic diseases	Toxicity
NCT04579523	^211^At	CD38	^211^At-OKT-B10 and fludarabine	Newly diagnosed, recurrent, or refractory high risk multiple myeloma	MTD
NCT04466475	^211^At	CD38	^211^At-OKT-B10 and melphalan	Relapsed or refractory multiple myeloma after at least 3 lines of prior therapy	MTD
NCT05363111	^225^Ac	CD38	^225^Ac-DOTA-daratumuab and daratumumab	Relapsed or refractory multiple myeloma after at least 2 lines of prior therapy	DLT, MTD
NCT05204147	^225^Ac	CEA	^225^Ac-DOTA-M5A	Metastatic solid tumors expressing CEA	AE, MTD

Abbreviations: AE, adverse events; DLT, dose-limiting toxicities; MTD, maximum tolerated dose; CEA, carcinoembryonic antigen.

## Data Availability

Not applicable.
